# Order of magnitude enhancement of monolayer MoS_2_ photoluminescence due to near-field energy influx from nanocrystal films

**DOI:** 10.1038/srep41967

**Published:** 2017-02-03

**Authors:** Tianle Guo, Siddharth Sampat, Kehao Zhang, Joshua A. Robinson, Sara M. Rupich, Yves J. Chabal, Yuri N. Gartstein, Anton V. Malko

**Affiliations:** 1 Department of Physics, The University of Texas at Dallas, Richardson, TX, 75080, USA; 2 Department of Materials Science and Engineering, The Pennsylvania State University, PA, University Park, 16802, USA; 3 Department of Materials Science and Engineering, The University of Texas at Dallas, Richardson, TX, 75080, USA

## Abstract

Two-dimensional transition metal dichalcogenides (TMDCs) like MoS_2_ are promising candidates for various optoelectronic applications. The typical photoluminescence (PL) of monolayer MoS_2_ is however known to suffer very low quantum yields. We demonstrate a 10-fold increase of MoS_2_ excitonic PL enabled by nonradiative energy transfer (NRET) from adjacent nanocrystal quantum dot (NQD) films. The understanding of this effect is facilitated by our application of transient absorption (TA) spectroscopy to monitor the energy influx into the monolayer MoS_2_ in the process of ET from photoexcited CdSe/ZnS nanocrystals. In contrast to PL spectroscopy, TA can detect even non-emissive excitons, and we register an order of magnitude enhancement of the MoS_2_ excitonic TA signatures in hybrids with NQDs. The appearance of ET-induced nanosecond-scale kinetics in TA features is consistent with PL dynamics of energy-accepting MoS_2_ and PL quenching data of the energy-donating NQDs. The observed enhancement is attributed to the reduction of recombination losses for excitons gradually transferred into MoS_2_ under quasi-resonant conditions as compared with their direct photoproduction. The TA and PL data clearly illustrate the efficacy of MoS_2_ and likely other TMDC materials as energy acceptors and the possibility of their practical utilization in NRET-coupled hybrid nanostructures.

Monolayer transition metal dichalcogenides (TMDCs) such as MoS_2_, MoSe_2_, WS_2_ constitute a new class of two-dimensional (2D) direct-gap^1^ semiconductors widely believed to be promising candidates for various optoelectronic applications^2^,^3^,^4^,^5^. Thanks to the 2D confinement and reduced dielectric screening, these systems support tightly-bound electron-hole pairs, excitons^6^, that are prominently featured in their light absorption^1^ and emission^7^ spectra. Like with other semiconductors, the common means to create excitons in monolayer TMDCs are via the direct photon absorption or via the injection of electrons and holes, which respectively lead to the phenomena of photoluminescence (PL)^7^ and electroluminescence^8^, as experimentally observed in these materials. It is known, however, that recombination losses^9^,^10^ of the excitations photoproduced in monolayer MoS_2_ can result in substantial limitations on the observed PL emission quantum yield (QY), which is commonly found well below 1% in the as-prepared samples^11^.

A very different way to produce excitons in TMDCs is through near-field coupling that enables nonradiative energy transfer (NRET) from the proximal quantum emitters such as photoexcited nanocrystal quantum dots (NQDs). NRET is a fundamental physical process well known for being one of the primary processes of photosynthesis^12^ and vital for energy transport in molecular systems^13^,^14^, and that can also be exploited in judiciously designed hybrid nanostructures for novel optoelectronic devices^15^,^16^,^17^. Excitonic sensitization via NRET should be contrasted with other commonly used schemes to enhance light absorption, such as via the plasmonic enhancement^18^,^19^. While the latter effect Strengthens the electric field in the vicinity of the monolayer absorber, the resulting excitons could undergo even larger nonradiative recombination losses leading to low QYs. On the other hand, excitons in NQDs are known to possess high QYs that are maintained in nanocrystal solids^20^, their radiative lifetimes are sufficiently long to allow for effective gradual energy transfer (ET) into the monolayer, which can potentially dramatically reduce the parasitic recombination losses.

Energy transfer from NQDs into TMDCs has attracted increasing attention recently^21^,^22^,^23^,^24^,^25^,^26^,^27^ and exhibits interesting physics due to the strong dielectric polarization response of TMDC systems and concomitant non-additivity^28^ of NRET rates. We predicted^28^,^29^ that NRET into highly-polarizable ultrathin semiconducting layers is, counterintuitively, more efficient for thinner rather than thicker layers, and that its distance *h* dependence should be slower than the traditionally^30^,^31^,^32^ assumed 1/*h*^4^ scaling. In experiments^21^,^26^ with MoS_2_, NRET from NQDs into a monolayer was indeed found more efficient than into few-layer samples. Our recent observation^25^ of quite high, 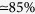, NRET efficiency from individual (arranged in sub-monolayers) CdSe/CdS NQDs with a large radius that defines the appreciable separation distance of 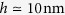 between NQDs and MoS_2_ monolayers gives further support to the idea of effective excitonic sensitization of TMDCs.

In this paper, we demonstrate that PL properties of monolayer MoS_2_ can be greatly enhanced in the hybrid nanostructure with optically thick films of CdSe/ZnS core-shell NQDs. We characterized and employed these NQDs previously in studies of ET in various configurations (ref. 20 and citations therein). Smaller-size CdSe/ZnS NQDs enable higher efficiency of NRET into a neighboring substrate and can potentially facilitate the internal energy transfer (spectral diffusion^33^,^34^,^35^) between the layers of nanocrystals towards MoS_2_ substrate. The novelty of our approach is to employ both ultrafast time-resolved PL and transient absorption (TA) pump-probe spectroscopies, which has not been done so far for these systems, to directly observe the dynamic evolution of excitonic signatures in monolayer MoS_2_ as it is accepting energy from the NQDs. While common to probe the dynamics of directly photoexcited species (including the effects of *charge transfer* in TMDC bi-layer structures^36^), the TA method has never been applied before to monitor the absorption changes of an energy acceptor in the process of *energy transfer*. Both PL and TA data derived on NQD/MoS_2_ hybrids exhibit nanosecond-scale kinetics absent in pristine MoS_2_, which is the time scale consistent with measured and evaluated NRET rates. The data unequivocally demonstrate highly effective excitonic sensitization of monolayer MoS_2_ in the hybrid structures as manifested by a nearly 10-fold enhancement of the MoS_2_ PL intensity in hybrid structures in comparison with the PL emission resulting from the direct photon absorption in the bare MoS_2_ reference monolayers. Our findings indicate that excitonic sensitization of TMDCs via ET occuring on a comparatively “slow”, nanosecond, time scale is capable of greatly reducing the recombination losses and dramatically increase the quantum yield of the emissive excitons in monolayer MoS_2_. The ET approach is thus expected to extend the range of optoelectronic application opportunities for monolayer dichalcogenide systems, further enhanced by the possibility of electric manipulation^22^ of the strength of near-field coupling.

## Sample Description and Experimental Setup

Monolayer MoS_2_ domains with sizes between 5–10 *μ*m were prepared by chemical vapor deposition (CVD) on top of transparent sapphire substrates to cover nearly uniformly ~cm^2^ of the substrate’s surface. The domains were grown by powder vaporization technique. 2 mg of MoO_3_ (99.8%, Sigma) was placed in an alumina crucible at the center of the furnace. 400 mg of S powder (99.995%, Alfa Aesar) was positioned 12 inch upstream from the MoO_3_ crucible. 270 nm SiO_2_/Si substrate (University Wafer) was located 7 mm away from the MoO_3_ powder, facing the powder by its glass surface. The growth temperature was 725 C for 15 minutes with a 10 minutes dwelling at 300 C in order to remove the organic/water residuals. After the growth, the furnace cooled down at a rate of 30 C/min until reached 300 C, then followed by a natural cooling. Monolayer thickness of the flakes was ascertained by Raman spectroscopy.

The synthesis of CdSe/ZnS NQDs with PL emission wavelength at ~585 nm was based on a well-established literature method^37^ with a minor modification: the injected volume of TOP was doubled. Core-shell NQDs with 2 CdS and 4 ZnS shells were prepared according to the SILAR technique^38^,^39^. Detailed information about crystal structure and elemental composition, as well as the size of resulting CdSe/ZnS NQDs is shown in Fig. S1 of the Supplementary Information (SI). Their absorption and emission spectra are presented in Fig. S2(a). This emission wavelength corresponds to NQD size of ~5.5 nm as determined by TEM (Fig. S1(c)). Together with ligands, the total size of NQDs is ~7.5 nm. The emission quantum yield of the NQDs in solution was measured using integrating sphere and found to be about 55% – in agreement with the results we reported in ref. 20.

To prepare hybrid samples, NQDs are drop-casted from hexane solution to form a dense coverage over substrates with MoS_2_. Numerous studies have shown that oleic acid ligands commonly used to passivate NQDs effectively prevent charge transfer to semiconductor substrates^40^,^41^,^42^. Instead, energy transfer has been shown to be the main mechanism leading to substrate sensitization from nearby quantum emitters, including TMDC substrates^21^,^22^,^24^,^25^,^26^,^27^. It should be emphasized that energy transfer is a much longer-range process than more conventional electron transfer^13^,^14^. The electron transfer at interfaces is mediated by the overlap of electronic wave functions and therefore can be greatly affected by the quality of the interface. Energy transfer, on the contrary, is mediated by the near-zone electric field that is practically not influenced by the details of the interface morphology (ref. 17 and citations therein) in the standard drop-cast deposition procedure. We estimate NQD film thickness of ~150 nm based on the calibrated linear absorption spectra of the self-assembled NQD layers^20^ as seen in Fig. S2(b). In this paper we do not pursue studies of the magnitude of the effects as a function of the thickness of NQD films.

Time-resolved pump-probe measurements are based on an amplified Ti:Sapphire laser system producing a fundamental beam with 100 fs pulses at 800 nm. Pump beam is produced by frequency doubling fundamental to 400 nm in the BBO nonlinear crystal, while smaller part of the fundamental beam is focused into sapphire plate to produce visible wight light continuum (WLC) probe in the region of 450–800 nm. All measurements are made at room temperature. Complementary time-resolved PL measurements of individual MoS_2_ domains are performed in Olympus IX 71 microscope equipped with nanopositioning x-y stages with step size resolution of 50 nm with the sample excited by 405 nm laser pulses of 50 ps duration at the repetition rate of 5–20 MHz. Time-tagged time-correlated single photon counting (TCSPC) is performed using PicoQuant TimeHarp 200 electronics. To compute pulse fluences, diameters of the excitation spot sizes have been measured, providing 200 *μ*m of the pump beam in TA experiment and 0.6 *μ*m in PL experiments. NQD occupation has been computed using tabulated absorption cross-section of 5 × 10^−15^ cm^−2^ as previously established. We monitor the PL emission and TA signatures of excitons in monolayer MoS_2_ that follow the excitation of the system (both NQDs and MoS_2_) by a laser pulse. Throughout the text, the laser excitation levels are indicated in the pulse fluence (mJ/cm^2^) as appropriate for MoS_2_ and in the corresponding number of electron-hole pairs, *N*_eh_, as common in the NQD spectroscopy.

## Results

### Order of magnitude enhancement of MoS_2_ PL emission in hybrids

**Figure 1:**
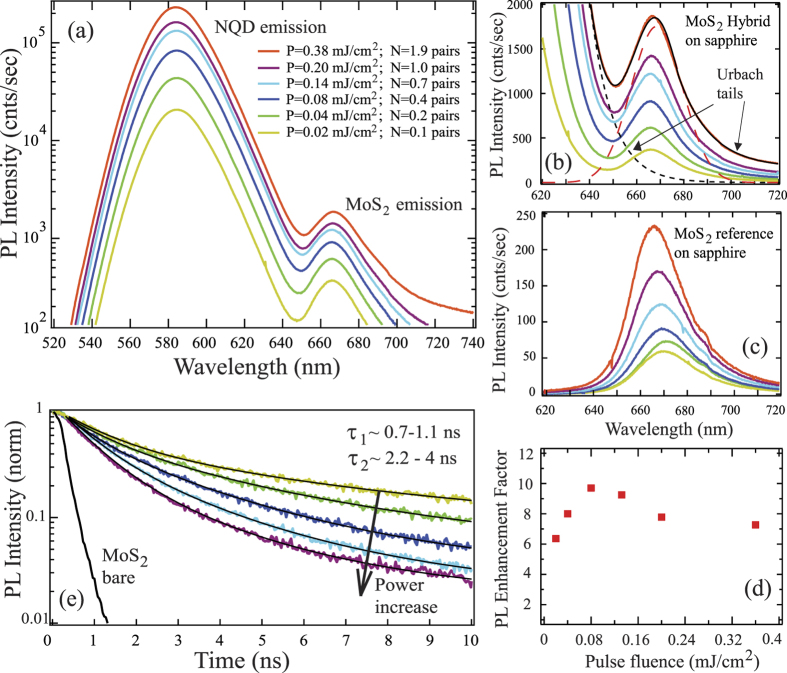
(**a**) PL intensity of NQD/MoS_2_ hybrid at several pump levels on the log scale. (**b**) Magnified view of the MoS_2_ PL emission region in the hybrid samples. Black solid line - fit of the top trace using Gaussian (red dashed line) for MoS_2_ PL peak and two exponentials for Urbach-like sub-band gap emission in NQDs (black dashed line) and in MoS_2_ (part of the fit line in 690–720 nm region) (**c**) PL of the bare monolayer MoS_2_ reference sample at the same power levels. The large increase of PL intensity is quantified in panel (d) in the form of the PL enhancement factor (ratio of MoS_2_ PL intensities in panels (b,c)) as a function of the pulse fluence. (**e**) Time-resolved PL decays for the hybrid at 676 nm fitted by double-exponentials and compared to the PL decay in the reference sample.

Figure 1 compares the PL spectra of the NQD/MoS_2_ hybrid and reference MoS_2_ samples at different pump fluence levels. The PL spectrum of the hybrid plotted in the logarithmic scale in Fig. 1(a) clearly shows a well-resolvable superposition of the emission from both components: the NQD emission centered at 585 nm and the luminescence from MoS_2_ at 676 nm. While the signal from NQDs is obviously much larger (due to significant light absorption and high quantum yield in the thick NQD film), the MoS_2_ emission is spectrally well separated at all pump levels. Panel (b) of Fig. 1 shows a magnified view of the MoS_2_ emission region in the hybrid sample. The top spectrum (red solid curve) was decomposed into the PL contribution from the MoS_2_ (red dashed Gaussian peak) and two exponential Urbach-like tails corresponding to sub-gap states in the NQD material (black dashed line) and in MoS_2_ itself (part of the solid black line fit in the 690–720 nm region). It is clear that NQD’s tail contribution to MoS_2_ PL at 676 nm is very small. A similar decomposition performed for spectra at lower pump powers shows even smaller contributions of NQD’s emission leakage to MoS_2_ region. Panel (c) displays reference PL emission spectra of monolayer MoS_2_ at the same power levels before NQD deposition. An order of magnitude PL enhancement effect of NQDs on the MoS_2_ photoluminescence is evident in Fig. 1(d) which shows the ratio of MoS_2_ PL intensity in hybrid (corrected for small amount of NQD’s tail emission leakage) to the reference MoS_2_ monolayer sample (that is, the sample *before* deposition of NQDs). Up to a 10-fold increase of the MoS_2_ emission is observed in the hybrid sample clearly implying efficient ET to monolayer MoS_2_ from a large number of photoexcited NQDs.

While the luminescence intensity increase from the acceptor material would ordinarily be considered a sufficient evidence of ET, such an increase could also be influenced by variations of the QY of the acceptor emission in the hybrid as we discussed above. We compared PL of the bare monolayer MoS_2_ before and after the exposure to the solution of organic ligands used for our NQDs and did not find noticeable differences (Fig. S3 of SI). However, given the complexity of surface passivation properties of MoS_2_ monolayers, ref. 11 a more direct observation of the time-dependent *process* of energy transfer is highly desirable. Further insight and evidence can correspondingly be obtained from the kinetics of the MoS_2_ luminescence, which is presented in Fig. 1(e). This figure compares the time-resolved MoS_2_ emission in the reference bare MoS_2_ sample with that in the hybrids at different excitation levels. We emphasize that the PL decay in the reference sample as observed here is limited by our PL system resolution, correspondingly the signal displays a nearly monoexponential behavior convoluted with the instrument response function of the detector with the response limited time ~0.3 ns. More precise measurements made in ref. 22 with a streak camera as well as our own much better time-resolved TA data indicate that bare MoS_2_ PL lifetimes are significantly shorter, ~10 ps. Even with this resolution limitation of the bare MoS_2_ signal, though, Fig. 1(e) already provides a transparent illustration of much slower luminescence decays in hybrid samples, overall on a nanosecond time scale. From the double-exponential fits, their faster components exhibit ~0.7–1.1 ns and slower components ~2–4 ns lifetimes. To rule out PL signal “contamination” at 676 nm by a small amount of NQD emission tail overlap, Fig. S4(a) of SI compares PL lifetimes at 676 nm for the hybrid sample and for the NQD-only sample similarly deposited on the sapphire substrate. It is very clear that the PL signal in the hybrid is orders of magnitude larger than the signal from the NQD-only sample, demonstrating that no signal contamination takes place at 676 nm from the NQD tail emission. We further note that the faster component of lifetime is comparable with the PL quenching time that we measured at 585 nm for the sub-monolayer NQD donors on MoS_2_ sample, which is a signature of ET on the donor side (Fig. S4 of SI). The data in Fig. 1(e) thus unequivocally show that the emission from MoS_2_ in the hybrids occurs on an extended time scale as determined by the dynamics of energy influx into the monolayer rather than by its intrinsic decay time. The pump-level dependence of the traces displayed in Fig. 1(e) is reflective of nonlinear (such as Auger-like or Auger-assisted) relaxation processes leading to saturation effects at higher pumping levels well-known in both NQDs and MoS_2_. We will refer to these effects later in this paper.

### Dynamics of the energy acceptor population in hybrid samples

While the energy supply from thick donor films may, generally speaking, exhibit a convoluted dispersive time dependence, it is instructive and helpful to examine the dynamics of the exciton population in the *simplest* kinetic description with only very few defined rates and without nonlinearities. In such a description, the temporal evolution of the donor, *N*_D_(*t*), and acceptor, *N*_A_(*t*), excitations is governed by a system of two kinetic equations:


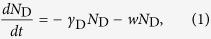



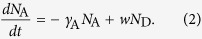


Here, *w* denotes the rate of ET from the excited donor to the acceptor, whereas *γ*_D_ and *γ*_A_ are the decay rates for the donor and acceptor, respectively, due to *other* radiative and nonradiative processes. Equations (1) and (2) readily yield


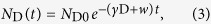



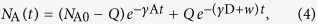


where *N*_D0_ and *N*_A0_ are the initial populations of the donor and acceptor excitations at time *t* = 0 and 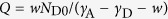. In this simplified description, one is understandably dealing with effective quantities: so *N*_D_ refers only to the donors that are well coupled to the acceptor subsystem while initial populations *N*_A0_ and *N*_D0_ to the actually available excitations that resulted from the direct photon absorption. As per Eqs (1) and (2), the total number of excitations transferred from the donor to acceptor is 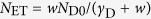 so that Eq. (4) can be rewritten as


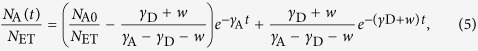


where *N*_ET_ is meaningfully compared to the initial number *N*_A0_ of the acceptor excitations. In accordance with the ET influx, the acceptor population (5) features a double-exponential behavior. The relationship between the two exponential terms, however, strongly depends on the interplay of the system parameters and initial conditions. For instance, we successfully applied^35^ this kinetic model to NQD bilayers in the regime of 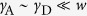, where ET resulted in the appearance of the rise-time behavior for the acceptor PL time evolution. In the case under consideration, however, with the MoS_2_ acceptor, the characteristic order-of-magnitudes of relevant times may be estimated as 1/*γ*_A_ ~ 10 ps, 1/*γ*_D_ ~ 10 ns, 1/*w* ~ 1 ns, so that the relationship between the rates is quite different:


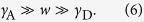


Equation (5) can, in principle, be used for the extraction of the salient system parameters and the ratio *N*_A0_/*N*_ET_ from the fits to the experimental data. In the parameter regime (6), the second term in Eq. (5) clearly illustrates that it is energy transfer with ET rate *w* that would be determining the longer-term decay of the number of acceptor excitations – after the initial excitations represented in the first term die off. This picture is in agreement with observations in Fig. 1(e) of much slower emission decays in hybrid samples. Given the limited time resolution of the PL measurements, however, the acceptor emission data in Fig. 1(e) are not the best candidates for accurate fitting with extremely short lifetimes 1/*γ*_A_. Instead, we are taking advantage of relationship (6) to employ ultrafast transient absorption measurements for addressing ET dynamics.

### Pump-probe study of the acceptor dynamics induced by ET from nanocrystals

In the investigation of energy transfer in hybrid systems, there are two sides of the process that can be looked at: the energy donor (NQDs in our case) and the energy acceptor (here monolayer MoS_2_). While the majority of experiments on NQD/TMDC hybrids have traditionally looked at the modification of the donor PL emission (energy *outflow*), the main focus of the study in this paper is distinctly different: on the side of the energy acceptor for both PL and TA measurements. This approach is especially important to analyze ET into MoS_2_ -like materials. Unlike energy donor-acceptor pairs with high emission quantum yields (QYs), where the donor emission quenching is well matched by the acceptor emission enhancement, monolayer MoS_2_ ordinarily exhibits very fast PL decays with low emission QYs^9^,^11^ so that the number of emissive excitons created in MoS_2_ can be substantially lower than expected on the basis of its absorptive properties alone. Low QYs may be related to the surface defects of TMDC materials and can vary with chemical surface passivation^11^. As hybrid structures contain organic ligands used to passivate NQD surfaces, those might inadvertently influence the emissive properties of MoS_2_ as well. The pump-probe TA spectroscopy relies on the modulation of absorption rather than emission and thus enables us to detect excitons that may not emit. Providing time resolution better than 1 ps, the TA pump-probe technique is thus introduced here as a novel method to directly visualize and quantify energy *influx*, particularly powerful for acceptors with fast intrinsic lifetimes, such as monolayer MoS_2_ under consideration.

**Figure 2:**
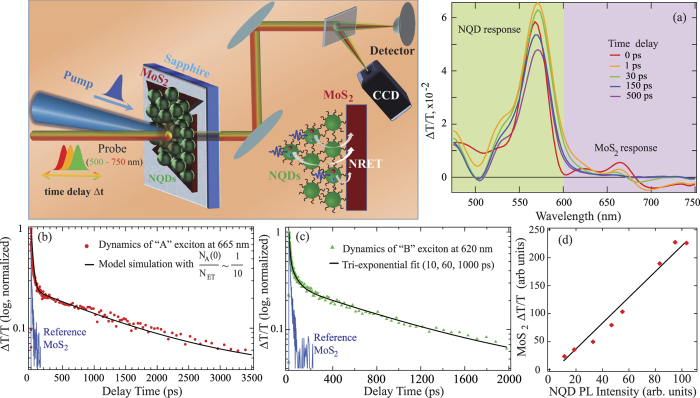
Schematic pictures of the pump-probe measurements setup and ET in hybrid NQD/MoS_2_ samples accompanied by the data plots. (**a**) Pump-probe spectra of the NQD/MoS_2_ hybrids for different time delays Δ*t* at ~1 mJ/cm^2^ pulse fluence. (**b**) Dynamics of the A-exciton bleaching feature in the hybrid (red trace) and reference MoS_2_ (blue trace) samples. Black line shows the fit using Eq. (5) yielding the parameter ratio 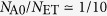. (**c**) Dynamics of the B-exciton bleaching feature in the hybrid (green trace) and reference MoS_2_ (blue trace) samples. Black line shows the fit by a tri-exponential function with 10, 60 and 1000 ps time constants. (**d**) The MoS_2_ exciton 660 nm bleach amplitude at Δ*t* = 150 ps in the hybrid sample vs the NQD PL intensity at 585 nm for the same pump fluences in the range of *N*_eh_ from 0.1 to 2.

Figure 2(a) shows differential transmission spectra Δ*T*/*T* of a hybrid NQD/MoS_2_ structure for several time delays Δt between the pump and probe pulses. As photoinduced TA signatures of both NQDs^43^,^44^ and monolayer MoS_2_^9^,^45^ have been well described, one immediately recognizes a superposition of their prominent bleaching (positive Δ*T*/*T*) features: at 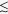
600 nm for NQDs and at 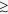
600 nm for MoS_2_. A clear spectral separation of longer-wavelength MoS_2_ TA features allows us to reliably compare their dynamics in hybrid and reference samples. More details on the TA signatures in our reference bare MoS_2_ samples are available in SI, Fig. S5; for a broader overview, we refer the reader to recent ref. 45. Here we concentrate on monolayer MoS_2_ bleaching features in the spectral regions of the so-called A-(monitored at 665 nm) and B-(monitored at 620 nm) excitons.

Figure 2(b) illustrates a drastic difference in the kinetics of the A-exciton bleaching signatures observed in the hybrid NQD/MoS_2_ and reference bare MoS_2_ samples. While the signal in the reference sample quickly dies off, the trace in the hybrid sample develops a very long “tail” with the decay time that is absolutely absent in the reference trace. If fitted with a three-exponential function, the hybrid traces exhibit components with lifetimes of ~1–2 ps, ~20–30 ps and ~1.5 ns. The determination of the latter lifetime may be a bit less precise here due to the relatively short observation window limited by the travel range of the delay line in the pump-probe experiments but it is evident that this component is about two orders of magnitude longer than any component in the reference MoS_2_ trace. In accordance with the picture presented in Eq. (5), the significantly extended decay of the MoS_2_ bleaching signal in the hybrid is reflective of the gradual energy influx from NQDs, which is much slower than the internal decay in MoS_2_: 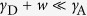.

Given the picosecond time resolution of the pump-probe experiments, the internal decay rate of the MoS_2_ can now be well resolved and the simplified fitting with the biexponential Eq. (5) attempted. For this fitting, we assume that the MoS_2_ bleaching signal is representative of the number *N*_A_ of the excitations in monolayer MoS_2_. Figure 2(b) shows a very satisfactory fit to the TA signal in the hybrid using Eq. (5) with time decay parameters 1/*γ*_A_ = 30 ps (in agreement with the decay in the reference sample), 1/(*γ*_D_ + *w*) ~ 1.7 ns and the ratio 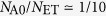 of the contributors to the exciton population in MoS_2_. The kinetics of the TA signal thus provides a direct confirmation of the effective ET time 1/*w* ~ 1.5 ns (in view of Eq. (6)) and clearly indicates very high efficacy of the excitonic “sensitization”: the number *N*_ET_ of excitons transferred to MoS_2_ from NQDs is about an order of magnitude larger than the effective number *N*_A0_ of excitons that resulted from the direct photoabsorption in MoS_2_. This conclusion corresponds rather well to the MoS_2_ emission enhancement in the hybrid samples observed in Fig. 1.

A drastic difference between the hybrid and reference samples is also observed in the dynamics of the bleaching feature in the spectral region of the MoS_2_ B-exciton. Figure 2(c) clearly illustrates the appearance of the long-lived tail in the hybrid with the decay time absent in the reference sample. Overall, the character of the changes in the hybrid is quite similar to what we observed for the A-exciton bleach in Fig. 2(b). Some variations are however also noticed. The kinetics of the B-exciton bleach in the hybrids can also be fit with tri-exponential functions, whose components exhibit both shorter lifetimes (~10–20 ps and ~50–100 ps) that are native to the MoS_2_, and new longer lifetimes ~600–900 ps due to ET from nearby NQDs. The ET times extracted from the fits to the bleach at 620 nm appear consistently shorter than ET times extracted from the fits to the bleach at 660 nm; nonetheless – and importantly – on the same time scale of ~1 ns. We also observed that the B-exciton bleach in hybrids exhibits more pump-power dependence than the A-exciton bleach. In view of these variations, it needs to be mentioned that A- and B-bleaching features display some differences already in bare monolayer MoS_2_, such as, for instance, a noticeable time-dependent spectral shift of the B-feature in Fig. S5 in SI. The possibility of excitation-energy dependent nonequlibrium populations of A- and B-excitons has also been discussed in the literature^46^.

It is useful to examine the pump-power dependence of the long-lived tail in the Δ*T*/*T* signals. The second term in Eq. (5) shows that the magnitude of this tail (that is, *N*_A_) should be proportional to *N*_ET_, and hence to the number *N*_D0_ of the NQD excitations created by the pump in the donor component of the hybrid. It is important to recall now that the number of the emissive excitons in NQD is not exactly proportional to the pump power: for high pump powers (*N*_eh_ > 1), the number of such excitons is well-known to tend to a saturated behavior due to nonlinear, non-radiative Auger recombination of multiexciton states in NQDs. The right measure of the relevant pump-dependent number of the excitons in NQDs is then their own PL signal. Figure 2(d) displays the magnitude of the bleach Δ*T*/*T* signal of the MoS_2_ A-exciton in hybrids at the delay time Δ*t* of 150 ps (at the beginning of the long tail in Fig. 2(b)) vs the PL intensity from NQDs at different pump powers. The resulting plot is evidently linear, clearly indicating that the long-lived signal in MoS_2_ is proportional to the number of excitons transferred from NQDs.

## Discussion

The data presented above provides unambiguous experimental evidence of highly effective excitonic sensitization of the light emission from monolayer MoS_2_ by means of energy transfer from the adjacent NQD film. The data shows that the number of excitons transferred from photoexcited NQDs into MoS_2_ is substantially larger than the number of observable excitons resulting from the direct optical absorption in monolayer MoS_2_. Indeed, on one hand we register a nearly 10-fold increase of the MoS_2_ emission in our hybrid structures. On the other hand, we observe that enhanced MoS_2_ excitonic signatures, in both luminescence and transient absorption, manifest themselves on an extended nanosecond timescale that is characteristic of the ET process and absent in stand-alone MoS_2_. The scope of our demonstration and the magnitude of the PL enhancement observed thus go well beyond a corresponding observation in ref. 22. We continue to elaborate to illuminate the likely origin of the effect that should allow for further application optimization.

### Light absorption and ET in hybrids

**Figure 3:**
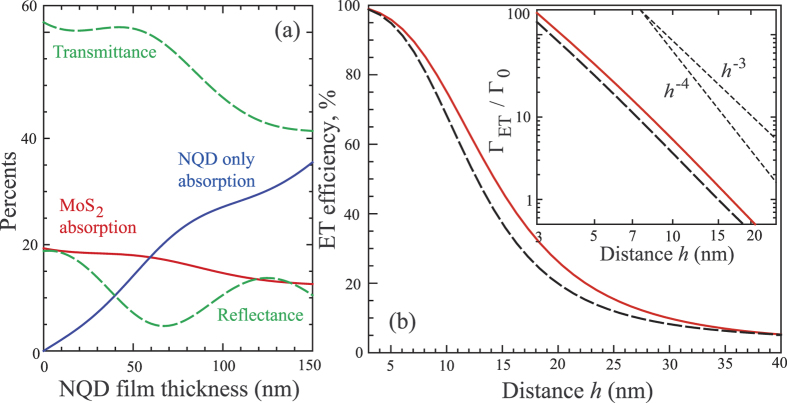
Results of illustrative model calculations using optical parameters discussed in refs 20 and 25. (**a**) Optical properties of the air/NQD/MoS_2_/sapphire/air structure for the 400 nm wavelength as a function of the NQD film thickness. Shown are the percentage amounts of the absorption in monolayer MoS_2_ and in the NQD film as well as the total reflectance and transmittance of the structure for the normally incident light. (**b**) The efficiency of energy transfer into MoS_2_ for the 585 nm wavelength as a function of distance *h* of randomly oriented electric-dipole emitter from the interface with monolayer MoS_2_. The inset in the double-logarithmic scale shows the distance dependence of the ET rates visually compared to power-laws of *h*^−3^ and *h*^−4^ displayed as short-dash lines. Solid lines show results for the interface between sapphire and vacuum, dash lines for the interface between sapphire and the medium with refraction index of 1.6 representative of dense NQD films^20^.

It is clear that excitonic sensitization of the acceptor subsystem should depend on such important factors as the number of excitons available in the donor subsystem and the efficacy of energy transfer between the subsystems. Our discussion of those factors for MoS_2_/NQD hybrids under consideration will be assisted by illustrative modeling results presented in Fig. 3. It should be noted that the precise values of optical parameters of pristine MoS_2_ are not firmly established yet as various measurements may be affected by samples’ preparation and nonuniformity. For the illustrative purposes here we use the frequency-dependent optical susceptibility of monolayer MoS_2_ extracted from our own transmittance measurements as described in ref. 25. The NRET process that we discuss in this paper has the same underlying physics as the well-known Förster energy transfer^13^,^14^ between small species that involves the overlap of the emission spectrum of the energy donor and the absorption spectrum of the energy acceptor. It has however its specific behavior as the energy transfer occurs into the spatially extended (two-dimensional in our case) acceptor material, whose collective response is represented by the corresponding complex-valued dielectric susceptibility at appropriate frequencies. Monolayer MoS_2_ susceptibility that we employ exhibits a highly absorptive behavior over a broad range of wavelengths, including in the overlap with the NQD emission wavelength used in this study. The emission-wavelength-dependence of ET into MoS_2_ was already illustrated and discussed in ref. 25. Optical parameters used for NQD films were derived from our ellipsometric measurements on multilayer NQD samples as reported in ref. 20.

Figure 3(a) compares the calculated amounts of the 400 nm laser excitation absorbed in monolayer MoS_2_ and in the NQD film as a function of the film thickness for the hybrid samples on thick sapphire substrates. (To appreciate the effect of MoS_2_ on the overall optical properties, see Fig. S2 of SI that compares the properties of the structures with and without monolayer MoS_2_). The calculations indicate that for NQD films of 100–150 nm thickness, the absorption in NQDs is expected to be comparable to that in reference monolayer MoS_2_, perhaps larger by a factor of 2 or so. With this in mind, the results of Fig. 1(a) showing the PL intensity from MoS_2_ – even after the enhancement in the hybrid – being about two orders of magnitude smaller than the PL intensity from NQDs should be interpreted as chiefly caused by very low emission QYs in monolayer MoS_2_, which can, according to the data in ref. 11, be well below 1%. On the contrary, our colloidal NQDs exhibit high PL quantum yields that, importantly, can be maintained upon formation of solid state films^20^. The excitons created in NQDs films can correspondingly live long enough to be able to undergo ET into MoS_2_.

Figure 3(b) shows the computed efficiency of ET as a function of the distance *h* from the excitonic NQD emitter (understood as from the center of the NQD) to interfacial monolayer MoS_2_ for the emission wavelength of 585 nm. The calculations were performed using the macroscopic electrodynamics framework for the decay of electric-dipole emitters in this geometric arrangement, the detailed description of which we provided in ref. 25. The efficiency of ET is defined here as the fraction Γ_ET_/Γ of the ET rate Γ_ET_ = Γ − Γ_rad_ in the total electrodynamic decay rate Γ of the emitter, when the purely radiative decay rate Γ_rad_ is subtracted. Figure 3(b) presents results derived for the interfaces between sapphire substrate and vacuum as well as between sapphire and the medium with refraction index 1.6 as representative^20^ of the dense NQD films, comparing which illustrates the extra screening effect on NRET that the film’s own polarizability could have (no account of relatively small local field effects^13^,^47^ was made here). As per Fig. 3(b), particularly high efficiencies of the direct ET events are restricted to the first few NQD monolayers adjacent to MoS_2_ (a single NQD monolayer can be estimated to add approximately 6 nm to the film thickness^20^). This is a consequence of the well-known strong dependencies of NRET on the donor-acceptor distances^31^,^32^, which is demonstrated for our case in the inset to Fig. 3(b) displaying Γ_ET_ in terms of the NQD vacuum radiative decay rate Γ_0_ (from our measurements of the NQD PL decay on glass surfaces, see Fig. S4 of SI, 1/Γ_0_ is estimated at ~24 ns). As a result of high polarizability^25^,^28^,^29^ of monolayer MoS_2_, the distance scaling here is still slower than *h*^−4^ conventionally assumed^30^,^31^,^32^ for the 2D acceptors. The strong distance dependence could generally result in a multitude of ET rates, from a fraction of a nanosecond into a nanoseconds range according to Fig. 3(b); those, however, may not be necessarily resolvable even with well-ordered NQD assemblies^20^ and all the more so with disordered NQD films. The overall dynamics of energy flows can further be complicated by the so-called spectral diffusion within the NQD film corresponding to the inter-dot energy transfer (see, e.g., refs 33, 34, 35 and Fig. S4(d) of SI). These considerations can account for certain variations of ET-related lifetimes derived with two- or three-exponential fits to different experimental traces from hybrids in Figs 1 and 2. It should be stressed then that all the lifetimes extracted from the fits to the dynamical traces of both emission and TA signals and featured only in the hybrid structures indeed fall into a sub-nanosecond to nanoseconds range, consistently with our expectations for energy transfer from NQDs into monolayer MoS_2_.

### Enhancement of MoS_2_ emission in hybrids

In trying to rationalize the enhancement of the MoS_2_ emission in hybrid samples, Fig. 1(d), it must be recognized that the number of transferred excitons is very improbable to be bigger than the number of photons directly absorbed in MoS_2_. Indeed, the latter number is expected to be comparable to the number of photons absorbed in the NQD film and only a fraction of the resulting excitons would be transferred, which is of course also reflected in a much larger emission signal from NQDs, Fig. 1(a). One, therefore, has to conclude that the effective emission QY from the transferred excitons should be appreciably higher than the QY of the excitations resulting from the photons directly absorbed in MoS_2_. In this paper we do not pursue measurements of the absolute values of the emission QY – similarly to other studies^10^,^22^, our experiments can target only the relative changes of the emission signal. Given the large number of the photons directly absorbed in MoS_2_, it is conceivable that the effective monolayer MoS_2_ emission QY could be increased in our hybrid nanostructure even more than the observed PL enhancement factors (Fig. 1(d)). The increase of quantum yields may be attributed to the substantial differences in the respective excitation modes.

First, the direct photon absorption in MoS_2_ takes place over the period of the laser pulse, ~100 fs, while energy transfer, in contrast, occurs over much longer, nanosecond-scale, times. The resulting momentary excitation density in monolayer MoS_2_ is thus much greater upon the direct photon absorption. The emission QY is known to be affected by both linear and nonlinear recombination processes. The linear regime would correspond to the decay channel(s) with the rates independent of the number of excitations. In the nonlinear regime, the recombination rates increase with the increase of the excitation densities, typical examples being Auger recombination^48^ and exciton-exciton annihilation^14^. Nonlinearity is manifested in the decrease of the QY for higher excitation powers and that is the type of behavior that is in fact observed^9^,^11^ in MoS_2_. In our own TA measurements on reference MoS_2_ samples, we clearly discern a strong non-linear response to the pump power already at the zero, Δ*t* = 0, delay time (Fig. S5 of SI), with Δ*T*/*T* signal only increasing twice when pulse fluence has been increased more than an order of magnitude. For the simplified kinetic description we discussed earlier in this paper, the nonlinear recombination effects at such short times will strongly reduce the effective number of the acceptor excitations, *N*_A0_. Thus, despite reasonable amount of optical absorption in MoS_2_ monolayer, the majority of directly absorbed photons do not result in optically active excitons. On the other hand, photoexcitation of nanocrystals results in production of a large number of long-lived excitons in NQD solids with high emission QY. The “gradual” delivery of these excitations from NQDs via the ET over nanosecond time scales results in much lower effective “pumping” fluence, avoiding non-linear Auger recombination and resulting in a much higher number, *N*_ET_, of optically active excitons in MoS_2_.

The second difference in the excitation modes is that the original laser pulse comes at the 400 nm wavelength, while energy transfer takes place at the NQD emission wavelength of 585 nm, which, respectively, may be labeled^46^ as “above-band gap” and “quasi-resonant” excitations for monolayer MoS_2_ with its large exciton binding energy. According to the studies in refs 10 and 46, these “distinctly different excitation methods”^46^ are accompanied by different relaxation dynamics and can lead to variances in the emission properties of monolayer MoS_2_. In particular, the quasi-resonant excitation is expected to lead to stronger A-exciton emission than the above-band gap excitation, which would result in substantially reduced A-exciton emission. The PL excitation spectra reported in ref. 10 explicitly show that this behavior is typical for a series of monolayer TMDC compounds. Interestingly, ref. 46 predicted luminescence even from B-excitons when MoS_2_ is excited quasi-resonantly. While our experimental data as well as the data in ref. 10 does not exhibit any emission from B-excitons, the enhancement of the MoS_2_ A-exciton emission in the hybrid samples is certainly consistent with the expectations for the quasi-resonant excitation, which is realized in our case by energy transfer from the NQD film.

## Conclusions

In summary, we have demonstrated that the excitonic sensitization of monolayer MoS_2_ via energy transfer (ET) from the adjacent NQD films in the NQD/MoS_2_ hybrid nanostructures results in a nearly 10-fold enhancement of MoS_2_ emission. This finding establishes ET as a distinct effective method to enhance PL in monolayer MoS_2_ and possibly other monolayer TMDCs. Energy transfer constitutes a qualitatively different way of excitation, whereby the conversion of the original optical pulses takes place both in terms of their time duration and excitation frequency. The absorbed energy of short pulses is delivered to MoS_2_ on an extended ET time scale that can dramatically lower the parasitic nonlinear recombination in monolayers. As the energy delivery occurs at the NQD emission frequency, the latter can be tuned to enable the appropriate optimal^10^ quasi-resonant excitation modality. Future studies should also allow for optimization of the NQD film thicknesses as appropriate for sensitization purposes. These findings and considerations are expected to increase the range of opportunities for optoelectronic applications of TMDC materials. Even more interesting physics of NQD/TMDC hybrids may be uncovered as experiments would be extending to low temperatures and fewer-defect samples, where NRET may result in the excitation of coherent exciton-polaritons in monolayers^23^, conceptually analogous to the excitation of surface plasmons by electric-dipole emitters^30^ observed in the proximity to metallic interfaces.

In addition to the specific results derived for the donor-acceptor system of interest in this paper, the focus and type of our studies can also be put in the context of a broader perspective of ET-based hybrid nanostructures^15^,^17^. While a body of literature exists that addresses ET from small donor species to various, including 2D, semiconductor acceptors, that research was done on the basis of the dynamics of donor emission quenching related thus to the energy *outflow* from the donors. Distinctly, in this paper we were able to approach and provide a first direct demonstration of the dynamics of the energy *inflow* into the semiconductor energy acceptor. We showed that the pump-probe transient absorption spectroscopy can be gainfully employed for studies of ET. This approach could, in particular, be used for studies of different energy acceptors that support non-emissive excitations, which would be undetectable by the traditional PL spectroscopy.

## Additional Information

**How to cite this article:** Guo, T. *et al*. Order of magnitude enhancement of monolayer MoS_2_ photoluminescence due to near-field energy influx from nanocrystal films. *Sci. Rep.* **7**, 41967; doi: 10.1038/srep41967 (2017).

**Publisher's note:** Springer Nature remains neutral with regard to jurisdictional claims in published maps and institutional affiliations.

## Supplementary Material

Supplementary Information
