# Supplementary material for: Order of magnitude enhancement of monolayer MoS2 photoluminescence due to near-field energy influx from nanocrystal films
Source: Sci Rep. 2017 Feb 3;7:41967. doi: 10.1038/srep41967 (PMC5290460; doi:10.1038/srep41967)
Supplement: Supplementary Information [file srep41967-s1.pdf]

SUPPLEMENTARY INFORMATION for Order of magnitude enhancement of monolayer MoS_2 photoluminescence due to near-field energy influx from nanocrystal films

Tianle Guo,[1] Siddharth Sampat,[1] Kehao Zhang,[2] Joshua A. Robinson,[2] Sara M. Rupich,[3] Yves J. Chabal,[3] Yuri N. Gartstein,[1] and Anton V. Malko[1,*]

[1]*Department of Physics, The University of Texas at Dallas, Richardson, TX, 75080, USA*

[2]*Department of Materials Science and Engineering, The Pennsylvania State University, PA, University Park, 16802, USA*

[3]*Department of Material Science, The University of Texas at Dallas, Richardson, TX, 75080, USA*

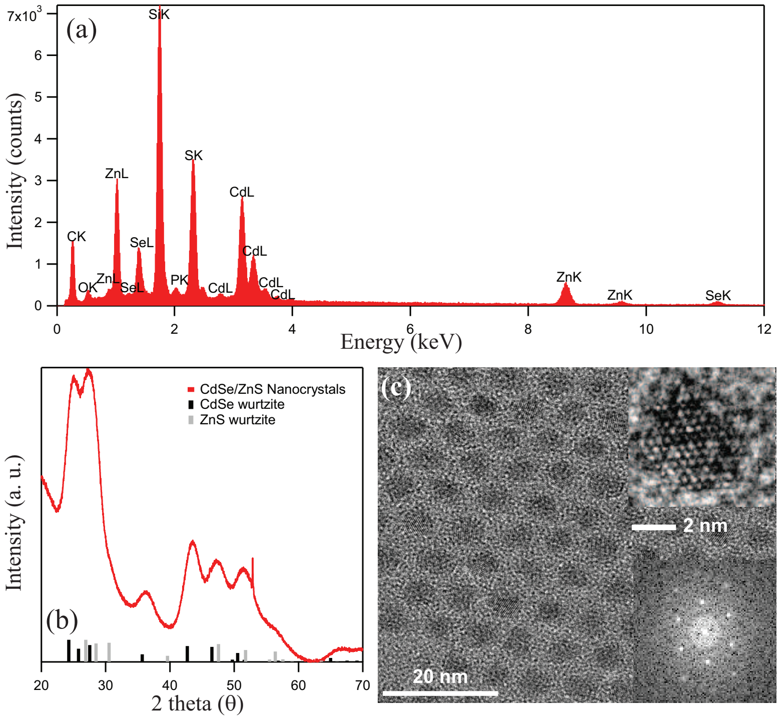

FIG. S1. (a)EDX data of the NQDs on Silicon showing their elemental composition. (b) GIXRD pattern of the CdSe/ZnS NQDs which correspond to a wurtzite crystal structure. Reference wurtzite patterns of bulk CdSe and ZnS are provided. (c) TEM image of sub-monolayer of NQDs deposited on Si surface. Average NQDs size $\sim$ 5.5 nm, total size including ligands $\sim$ 7.5 nm. Insets: HRTEM and its Fast Fourier Transform.

Comments for Figure S1.

EDX data was collected on a Zeiss Supro 40 equipped with an EDX detector. XRD data was collected on a Rigaku Smartlab. TEM images were collected on a Joel JEM-2100F Analytical TEM.

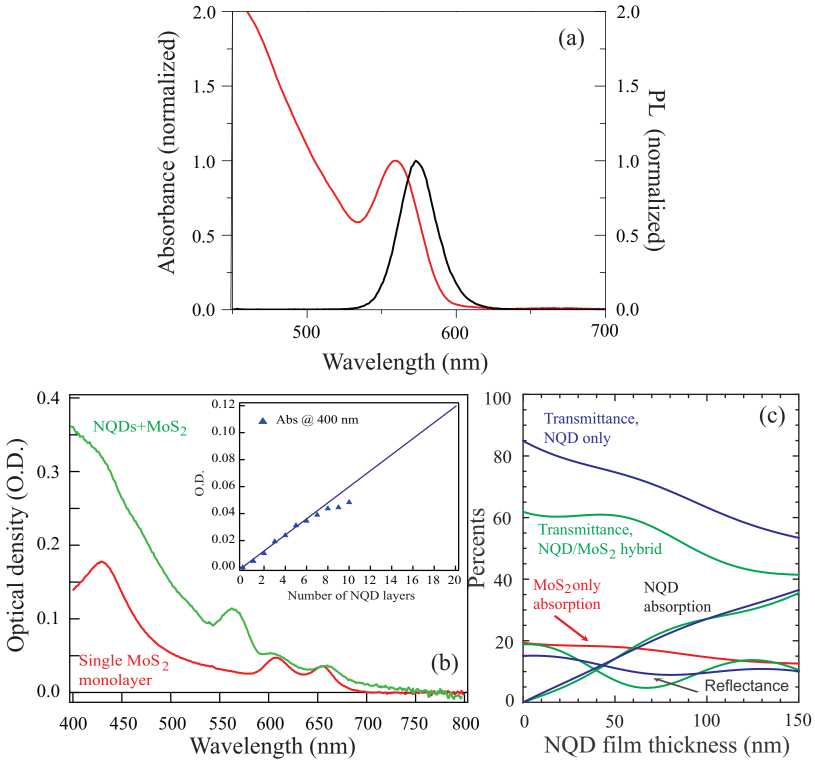

FIG. S2. (a) Normalized absorption (red) and emission (black) spectra of CdSe/ZnS NQDs in solution. (b) Absorbance spectra of a single MoS_2 monolayer flake (red) and a typical absorbance spectra of the drop-cast NQD film on top of MoS_2 (green). Inset: Optical density of a calibrated number of NQD monolayers deposited sequentially in a controlled manner on a glass substrate via self-assembly technique as per Ref. 1. Blue line: extrapolated absorbance using linear part of the trace. (c) Results of model calculations of optical properties (wavelength 400 nm) of stratified structures with a variable thickness of the NQD film using parameters discussed in Refs. 1 and 2. The percentages of transmittance, reflectance and component absorption for the normally incident light are compared for the structures with (air/NQD/MoS_2/sapphire/air, green and red lines) and without (air/NQD/sapphire/air, blue lines) monolayer MoS_2.

Comments for Figure S2.

In order to estimate the thickness of the NQD drop-cast films, we measured optical densities (O. D.) of the monolayer MoS_2 and the NQD/MoS_2 hybrid on our thick sapphire substrates. Drop-cast films typically do not form a uniform thickness layer, however, O. D. of the hybrid in the central region of the sample (where PL and TA measurements were made)

was found to be fairly uniform, with the representative O. D. spectra shown in Fig. S2(b). In our earlier work[1] with the same NQDs we were able to deposit a well-controlled number of dense NQD monolayers (MLs) on top of the glass substrate via the sequential self-assembly technique at air-liquid interface. The inset in Fig. S2(b) shows the resulting variation of O. D. at 400 nm as a function of number of MLs up to 10 NQD ML dense film. The measured O. D. apparently increases linearly, except for the very last few layer additions due to the incomplete layer formation. Following the linear extrapolation of that data indicates that NQD film O. D. of $\sim 0.15 - 0.20$ for the 400 nm excitation would correspond to the film thickness of about $25 - 30$ MLs. This range of NQD O. D. is actually what we typically observed in our hybrid samples (roughly, the difference between the green and red curves in Fig. S2(b)). The studies in Ref. 1 established the average NQD ML thickness of ~ 6 nm, corroborated by TEM measurements shown in Fig. S1(c). We can thus estimate the typical NQD film thickness in the region of the measurements around $150 - 180$ nm. One, however, understands that these values are approximate as subject to local variations in the disordered drop-cast films.

It is also helpful to put the discussed O. D. in the context of model computations of optical properties of our layered structures that were presented in Fig. 3(a) of the main text. Figure S2(c) compares the results of computations at 400 nm wavelength for the structures with and without monolayer MoS_2, thereby accentuating the effect of the monolayer on the overall optical properties. We employ the same set of optical parameters extracted from the experimental data in Refs. 1 and 2. The computational data in Fig. S2(c) yields the transmittance of the substrate at $T_0 \simeq 85\%$ (the left end of the blue curve) and the transmittance of the substrate with just monolayer MoS_2 at $T_1 \simeq 62\%$ (the left end of the green curve). The O. D. of the monolayer would then be $\log_{10}(T_0/T_1) \simeq 0.14$. On the other hand, the transmittance of the hybrid on the substrate with the 150 nm thick NQD film is computed at $T_2 \simeq 41\%$ (the right end the green curve), yielding the O. D. as $\log_{10}(T_0/T_2) \simeq 0.31$. The computed optical densities evidently agree rather well with the experimental data at 400 nm in Fig. S2(b), particularly given uncertainties involved.

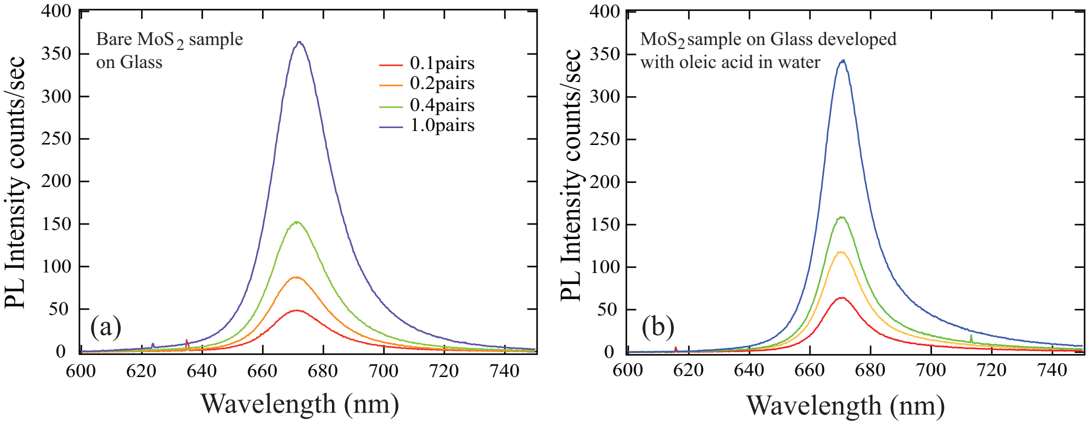

FIG. S3. (a) PL of the bare MoS_2 monolayers on glass substrate for different excitation levels; (b) PL of the same MoS_2 sample after incubation in the water solution with the oleic acid.

Comments for Figure S3.

It was reported[3] that PL properties of MoS_2 monolayers can be substantially affected by chemical surface passivation. Since NQD surfaces are passivated with oleic acid ligands, it needs to be verified if the PL of MoS_2 may be affected, when exposed to these organic agents. Fig. S3 compares the PL emission spectra of MoS_2 flakes on the glass substrate before, panel (a), and after, panel (b), a treatment with the water solution of the oleic acid. Evidently, both panels show nearly identical PL intensity in those samples, thus strongly indicating that MoS_2 surface/PL properties per se remain intact in the proximity of NQD films in our hybrid samples.

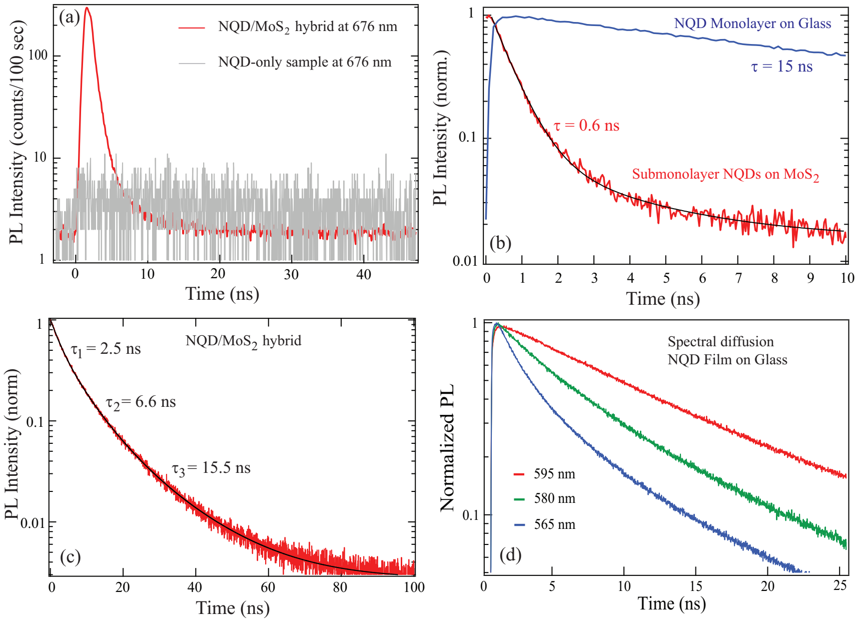

FIG. S4. (a) Comparison of the PL emission signal at 676 nm (central position of MoS_2 emission peak) for NQD/MoS_2 hybrid (red trace) and NQD-only sample deposited on a sapphire substrate for reference. (b-c) Time-dependent decays of the NQD PL measured at the peak NQD emission wavelength of 585 nm and the extracted PL lifetimes as indicated. Panel (b) compares the PL decays in (sub)monolayer NQD films deposited on the glass substrate and on a MoS_2 monolayer flake. Panel (c) illustrates the multi-exponential decays observed in the NQD/MoS_2 hybrids with thick NQD films.

Comments for Figure S4.

As we discussed in a number of earlier publications,[2,4–7] when NQDs are deposited in the form of a (sub)monolayer film on different substrates, the conditions are much more spatially uniform and the NQD PL decay can be quantified quite accurately in the relationship to the dielectric/optical properties of the environment. Figure S4(b) displays the reference PL decay of our NQDs on the glass substrate with the lifetime $\tau \sim 15$ ns, which, according to our analysis,[4–6] would translate in the vacuum PL lifetime $1/\Gamma_0 \sim 24$ ns (Γ_0 is the decay rate in vacuum). On the other hand, the second curve in Fig. S4(b) shows the greatly accelerated, to $\tau \sim 0.6$ ns, decay of the same NQDs in the vicinity of monolayer MoS_2. This acceleration is due to nonradiative energy transfer (NRET) into MoS_2 as discussed in the main text and quantified in its Fig. 3(b). As NRET is very strongly distance-dependent, the decay of NQDs in thick (and disordered) drop-cast films in our hybrid samples is expected to exhibit a very

dispersive behavior. This is indeed what we observed in such films: Fig. S4(c) illustrates the corresponding PL decay, which in this illustration was fit by a three-exponential with variable lifetimes. Moreover, as discussed in the main text, optically thick NQD films exhibit *internal* NRET between the NQDs themselves, with excitons migrating from smaller NQDs (emitting at the blue side of the PL emission spectrum) to larger and larger NQDs (emitting towards the red side of the spectrum). Fig. S4(d) illustrates this type of spectral diffusion for drop cast NQD film, with PL lifetimes at the blue side of the emission spectrum being considerably faster and multiexponential in nature.

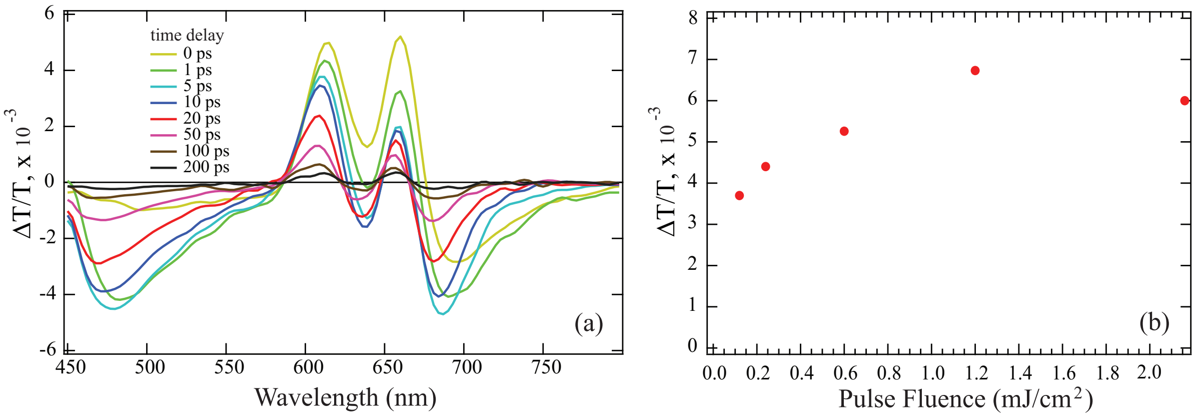

FIG. S5. (a) Transient absorption spectra of the reference monolayer MoS_2 sample for different time delays Δt between the pump and probe beams at 0.6 mJ/cm^2 pulse fluence. (b) The maximum of the bleach signal (wavelength 660 nm) at $\Delta t = 0$ as a function of the excitation pulse fluence.

Comments for Figure S5.

Transient absorption dynamics of monolayer MoS_2 was reported to exhibit a highly non-linear power dependence due to very fast Auger-associated recombination.[8] This is indeed the behavior we observe in our samples. Figure S5(a) shows pump-probe transients taken at 0.6 mJ/cm^2 pulse fluence, which display a very fast, $\lesssim 1$ ps, initial decay followed by a slower, $\sim 10 - 20$ ps, dynamics. The very fast non-linear recombination is further evidenced in Fig. S5(b), showing the pump fluence dependence of the maximum of the A-exciton bleach, that is, already at the *zero time delay*. It is clear that the amplitude of the $\Delta T/T$ signal in Fig. S5(b) varies no more than by a factor of 2 while the pulse fluence changes by more than an order of magnitude. We thus conclude that the pulse fluences in the range $0.1 - 1$ mJ/cm^2 used in this work result in the saturated behavior already at the shortest measurement times. In terms of the simplified kinetic model discussed in the main text, this means that the parameter N_{A0} of the initial number of the acceptor excitations is actually much lower than the number of the excited electron-hole pairs in MoS_2 that would correspond to its direct optical absorption.

* anton.malko@utdallas.edu

[1] Rupich, S. M., Gartstein, Y. N., , Malko, A. V. & Chabal, Y. J. Controlled deposition and spectroscopic signatures of ordered multilayer nanocrystal assemblies for optoelectronic applications. *Adv. Optical Mater.* **4**, 378–383 (2016).

[2] Sampat, S. *et al.* Exciton and trion energy transfer from giant semiconductor nanocrystals to MoS_2 monolayers. *ACS Photon.* **3**, 708–715 (2016).

[3] Amani, M. *et al.* Near-unity photoluminescence quantum yield in MoS_2. *Science* **350**, 1065–1068 (2015).

[4] Nguyen, H. M. *et al.* Spectroscopic evidence for nonradiative energy transfer between colloidal cdse/zns nanocrystals and functionalized silicon substrates. *Appl. Phys. Lett.* **98**, 161904/1–3 (2011).

[5] Nguyen, H. M. *et al.* Efficient radiative and nonradiative energy transfer from proximal cdse/zns nanocrystals into silicon nanomembranes. *ACS Nano* **6**, 5574–5582 (2012).

[6] Nimmo, M. T. *et al.* Visible to near-infrared sensitization of silicon substrates via energy transfer from proximal nanocrystals: Further insights for hybrid photovoltaics. *ACS Nano* **7**, 3236–3245 (2013).

[7] Nguyen, H. M., Seitz, O., Gartstein, Y. N., Chabal, Y. J. & Malko, A. V. Energy transfer from colloidal nanocrystals into si substrates studied via photoluminescence photon counts and decay kinetics. *J. Opt. Soc. Am. B* **30(9)**, 2401–2408 (2013).

[8] Wang, H., Zhang, C. & Rana, F. Ultrafast dynamics of defect-assisted electron-hole recombination in monolayer MoS_2. *Nano Lett.* **15**, 339–345 (2015).
